# Robust AUC optimization under the supervision of clean data

**DOI:** 10.1038/s41598-024-66788-2

**Published:** 2024-07-19

**Authors:** Chenkang Zhang, Haobing Tian, Lang Zhang, Pengju Jiao

**Affiliations:** China Mobile (Suzhou) Software Technology Company Limited, Suzhou, 215163 China

**Keywords:** Computer science, Software

## Abstract

AUC (area under the ROC curve) is an essential metric that has been extensively researched in the field of machine learning. Traditional AUC optimization methods need a large-scale clean dataset, while real-world datasets usually contain massive noisy samples. To reduce the impact of noisy samples, many robust AUC optimization methods have been proposed. However, these methods only use noisy data and ignore the effect of clean data. To make full use of clean data and noisy data, in this paper, we propose a new framework for AUC optimization which uses clean samples to guide the processing of the noisy dataset based on the technology of self-paced learning (SPL). Innovatively, we introduce the consistency regularization term to reduce the negative impact of the data enhancement technology on SPL. Traditional SPL methods usually suffer from the high complexity of alternately solving the two critical sub-problems with respect to sample weights and model parameters. To speed up the training process, we propose a new efficient algorithm to solve our problem, which alternately updates sample weights and model parameters with the stochastic gradient method. Theoretically, we prove that our new optimization method can converge to a stationary point. Comprehensive experiments demonstrate that our robust AUC optimization (RAUCO) algorithm holds better robustness than existing algorithms.

## Introduction

The ROC (receiver operating characteristic) curve plots the true positive rate against the false positive rate as the decision threshold is varied^1^, and AUC (area under the ROC curve) generally measures the probability of a randomly drawn positive sample having a higher decision value than a randomly drawn negative sample^2^. In recent years, AUC has garnered significant attention and has been widely applied in real-world scenarios, such as medical diagnosis^3,4^, fraud detection^5,6^, information retrieval^7,8^ and so on. Moreover, because of the high complexity of the AUC optimization problem, many efforts have been devoted to developing efficient algorithms, such as batch learning methods^9–11^ and online learning methods^12,13^.

Real-world datasets often contain a substantial number of noisy samples, which can significantly impact the performance of machine learning methods. To tackle this challenge, various robust AUC optimization algorithms have been developed. Specifically, Yuan *et al.*^14^ introduced a groundbreaking loss function that exhibits greater resilience to noisy samples, all while retaining the efficiency advantage. Ren *et al.*^15^ proposed an AUC maximization algorithm that is impervious to noisy samples by combining feature analysis and outlier handling. Furthermore, they validated the algorithm through theoretical results. In addition, Gu *et al.*^16^ introduced the BSPAUC algorithm, which is rooted in a statistical objective. This algorithm harnesses the power of SPL technology to exclude noisy samples during the training process. Nonetheless, it is worth noting that these robust AUC optimization algorithms often overlook the utilization of a small set of clean samples that are readily available in practical scenarios^17–19^.

**Table 1:** Representative noise-robust algorithms. ($n, n_{\mathscr {B}}$ mean the number of all samples and min-batch samples respectively, and we have $n \gg n_{\mathscr {B}}$ and $n > n^2_{\mathscr {B}}$.).

Algorithm	Technology against noisy samples	AUC optimization	Using clean samples	Easing the impact of data enhancement	Time complexity of SPL method
BAUC-OF^15^	Outlier detection	$\checkmark$	$\times$	–	–
MTLCA^20^	Robust network	$\times$	$\checkmark$	–	–
DGKG^19^	Label correction	$\times$	$\checkmark$	–	–
SPBL^21^	SPL	$\times$	$\times$	$\times$	*O*(*Tn*)
SPRL^18^	SPL	$\times$	$\checkmark$	$\times$	*O*(*Tn*)
BSPAUC^16^	SPL	$\checkmark$	$\times$	$\times$	$O(Tn^2)$
RAUCO	SPL	$\checkmark$	$\checkmark$	$\checkmark$	$O(Tn^2_{\mathscr {B}})$

Actually, some robust studies have made efforts to make full use of both clean data and noisy data in classification problem. For example, SPRL^18^ is applied to estimate the noisy data under the supervision of the small set of clean data by the feedback of the loss function. Li *et al.*^19^ develop a new framework to correct the noisy labels by leveraging the knowledge learned from available clean samples and semantic knowledge graphs. Instead of learning visual representations directly, Veit *et al.*^20^ utilize a limited number of clean samples to learn a mapping between noisy labels and clean labels. Although many robust classification methods employ available clean samples to deal with the noisy dataset, to the best of our knowledge, this work is the first robust AUC optimization study using such clean samples as shown in Table 1.

To take full advantage of the available clean data and noisy data, in this paper, we propose a new framework for AUC optimization by using the self-paced learning (SPL) technology^22^. A typical SPL model tries to minimize a sum of weighted sample losses, where the weight is negatively related to the loss value of the corresponding sample. In this case, SPL can eliminate noisy samples with excessive losses from the training by gradually reducing their weights to 0. Inspired by the above, in this paper, we propose our new AUC optimization objective function which employs SPL technology to eliminate noisy samples under the supervision of available clean samples. Different from traditional SPL, we introduce the consistency regularization term to reduce the negative impact of the data enhancement technology on SPL. Traditional SPL methods need to alternately solve the two critical sub-problems with respect to sample weights and model parameters, which is obviously time-consuming. To speed up the training, we propose a new efficient algorithm, named RAUCO, which updates the weight and model parameters alternately by using the stochastic gradient method. Theoretically, we prove that our proposed RAUCO can converge to a stationary point. Comprehensive experiments demonstrate that our RAUCO algorithm holds better robustness than existing algorithms.

The main contributions of this paper are summarized as follows:

- We propose a new framework for AUC optimization which takes full advantage of the available clean data and noisy data. We also introduce the consistency regularization term to reduce the negative impact of the data enhancement technology on SPL, which has been neglected for a long time.
- We propose a new efficient algorithm, named RAUCO, which updates the weight and model parameters alternately by using the stochastic gradient method. Compared with the traditional SPL methods, which need to alternately solve the two critical sub-problems with respect to sample weights and model parameters, our proposed method has lower time complexity.
- Under mild assumptions, our theoretical results demonstrate that our method can converge to a stationary point. Experimental results demonstrate the superiority of our RAUCO algorithm in robustness.

## Preliminaries

In this section, we give a brief review of AUC optimization and self-paced learning.

### AUC optimization

Let $\{\mathbf{x}^+_i\}_{i=1}^{n^+}, \mathbf{x}\in \mathbb{R}^d$ be the set of positive samples labeled as $y = +1$, and $\{\mathbf{x}^-_j\}_{j=1}^{n^-}, \mathbf{x}\in \mathbb{R}^d$ be the set of negative samples labeled as $y = -1$. Existing AUC optimization studies consider the following AUC definition that is equivalent to the Wilcoxon-Mann-Whitney statistic^23,24^:

$$\begin{aligned} AUC(f_{\theta })=\text {Pr}(f_{\theta }(\mathbf{x}) \ge f_{\theta }(\mathbf{x}') | y=+1,y'=-1) = \mathbb{E}[ \mathbb{I}(f_{\theta }(\mathbf{x}) - f_{\theta }(\mathbf{x}') \ge 0 ) | y=+1,y'=-1 ], \end{aligned}$$

where $f_{\theta }: \mathbb{R}^d \rightarrow \mathbb{R}$ is the machine learning model and $\theta$ means the model parameters. $\mathbb{I}(\cdot )$ is the indicator function such that if *a* is true, $\mathbb{I}(a)$ equals to 1; otherwise, it is 0.

The above AUC definition $AUC(f_{\theta })$ demonstrates that AUC actually measures the probability of a randomly drawn positive sample having a higher decision value than a randomly drawn negative sample^2^. Considering that $AUC(f_{\theta })$ is composed of non-convex terms, directly maximizing $AUC(f_{\theta })$ yields a difficult optimization problem. To address this issue, the indicator function $\mathbb{I}(\cdot )$ is usually replaced by the surrogate convex loss, *e.g.*, hinge loss. In this case, many studies^10,11,16^ usually minimize the following AUC optimization objective instead:

1 $$\begin{aligned} \min _{\theta } \sum _{i=1}^{n^+} \sum _{j=1}^{n^-} \max \{ 1- f_{\theta }(\mathbf{x}^+_i) + f_{\theta }(\mathbf{x}^-_j), 0 \}, \end{aligned}$$

where $\max \{1-f_{\theta }(\mathbf{x}^+_i)+f_{\theta }(\mathbf{x}^-_j), 0 \}$ is the popular AUC hinge loss.

### Self-paced learning

Humans and animals often comprehend examples that are not randomly presented but organized in a meaningful order. Inspired by this, Kumar *et al.*^22^ propose a learning strategy called self-paced learning (SPL), which starts from learning samples with minor losses and then gradually trains samples with larger losses.

Let $\{(\mathbf{x}_i,y_i)_{i=1}^n\}$ be the training set, where $y_i\in \{+1,-1\}$ is the corresponding label of the sample $\mathbf{x}_i \in \mathbb{R}^d$. Considering that SPL assigns the weight to each sample according to the loss value, we formulate the sample weight vector as $\mathbf{w}\in [0,1]^n$. In this case, the SPL objective function for the binary classification problem is formulated as follows:

2 $$\begin{aligned} \min _{ \theta , \mathbf{w}\in [0,1]^n} \sum _{i=1}^n w_i L_{\text {binary}}(f_{\theta }(\mathbf{x}_i),y_i) + R(\mathbf{w},\lambda ), \end{aligned}$$

where $f_{\theta }: \mathbb{R}^d \rightarrow \mathbb{R}$ is the machine learning model, $\theta$ means the model parameters, $L_{\text {binary}}$ represents one binary classification loss, *e.g.*, hinge loss, $\lambda$ is the age parameter that controls the learning pace in SPL and $R(\mathbf{w}, \lambda )$ means the self-paced regularization term that influences the assignment of sample weights. Note that $R(\mathbf{w}, \lambda ) = - \lambda \sum _{i=1}^n w_i$ is one frequently-used self-paced regularization term.

The alternative optimization strategy^18,25,26^ is always applied to solve the SPL objective function. Specifically, we would alternatively minimize the following two critical sub-problems with respect to model parameters $\theta$ and sample weights $\mathbf{w}$:

3 $$\begin{aligned} \min _{\theta } \sum _{i=1}^n w_i L_{\text {binary}}(f_{\theta }(\mathbf{x}_i),y_i), \ \ \min _{\mathbf{w}\in [0,1]^n } \sum _{i=1}^n w_i L_{\text {binary}}(f_{\theta }(\mathbf{x}_i),y_i) + R(\mathbf{w},\lambda ). \end{aligned}$$

Significantly, the sub-problem with respect to model parameters $\theta$ can be considered as a weighted version of the original problem and can be optimized through the common practice of the original problem.

## Proposed algorithm

In this section, we first introduce our objective function and then propose our optimization algorithm.

### Objective function

As we explained earlier, our algorithm takes full use of both clean data and noisy data. In this case, we consider that samples are provided in two parts with different quantities: a small set of clean data $\{ \widetilde{\mathbf{x}}^+_i, \widetilde{\mathbf{x}}^-_j \ | \ i \in [m^+], j \in [m^-] \}$ and a large-scale noisy dataset $\{ {\mathbf{x}}^+_i, {\mathbf{x}}^-_j \ | \ i \in [n^+], j \in [n^-] \}$, where $\mathbf{x}\in \mathbb{R}^d$ and $m= m^+ + m^-, n^+ + n^- =n, m \ll n$. Also, $f_{\theta }: \mathbb{R}^d \rightarrow \mathbb{R}$ is the machine learning model and $\theta$ means the model parameters. For the noisy dataset, let $\mathbf {w^+}\in [0,1]^{n^+}, \mathbf {w^-}\in [0,1]^{n^-}$ be the sample weight vectors of positive samples and negative samples. Moreover, $\mathscr {T}(\mathbf{x})$ means one data enhancement version of the sample $\mathbf{x}$, such as scaling, rotating, shearing, flipping and so on^27^.

Here, our objective function is formulated as follows:

4 $$\begin{aligned} \mathscr {L}_{\lambda }(\theta ,\mathbf{w}) =&\underbrace{ \tau ||\theta ||^2 }_{\mathbf{1}} \underbrace{ + \gamma ||\theta -\widetilde{\theta }^*||^2}_{\mathbf{2}} \underbrace{ + \sum _{i=1}^{m^+} \sum _{j=1}^{m^-} \frac{ \xi (\widetilde{\mathbf{x}}^+_i,\widetilde{\mathbf{x}}^-_j) }{m^+ m^-} }_{\mathbf{3}} \underbrace{ + \sum _{i=1}^{n^+}\sum _{j=1}^{n^- } \frac{ w_i^+ w_j^- \xi ({\mathbf{x}}^+_i,{\mathbf{x}}^-_j) }{n^+ n^-} }_{\mathbf{4}} \underbrace{- \lambda \left( \sum _{i=1}^{n^+} \frac{w_i^+}{n^+} +\sum _{j=1}^{n^-} \frac{w_j^-}{n^-} \right) }_{\mathbf{5}} \nonumber \\&\underbrace{+\, \mu \left( \sum _{i=1}^{n^+} \frac{w^+_i}{n^+} -\sum _{j=1}^{n^-} \frac{w^-_j}{n^-} \right) ^2}_{\mathbf{6}} \underbrace{+ \rho \left( \sum _{i=1}^{n} \frac{\zeta (\mathbf{x}_i)}{n} +\sum _{j=1}^{m} \frac{ \zeta (\widetilde{\mathbf{x}}_j) }{m} \right) }_{\mathbf{7}} \nonumber \\&s.t.\ \ m=m^+ + m^-, n=n^+ + n^-, \mathbf{w}\in [0,1]^n, \xi (\mathbf{x}^+,\mathbf{x}^-)=\max \{ 1-f_{\theta }(\mathscr {T}(\mathbf{x}^+))+f_{\theta }(\mathscr {T}(\mathbf{x}^-)), 0 \}, \nonumber \\&\qquad \underbrace{ \widetilde{\theta }^* = \mathop {\mathrm {arg\,min}}\limits _{\theta } \tau ||\theta ||^2 + \sum _{i=1}^{m^+} \sum _{j=1}^{m^-} \frac{ \xi (\widetilde{\mathbf{x}}^+_i,\widetilde{\mathbf{x}}^-_j) }{m^+ m^-}}_{\mathbf{8}}, \underbrace{\zeta (\mathbf{x})=|| f_{\theta }(\mathbf{x}) - f_{\theta }(\mathscr {T}(\mathbf{x})) ||^2}_{\mathbf{9}}. \end{aligned}$$

For the above formulation, item 1 is the model regularization term to avoid overfitting, items 2 and 8 expect that the difference between the current model and the model obtained from training clean samples would be insignificant. Also, items 3 and 4 represent the AUC loss of the clean dataset and the noisy dataset. Moreover, item 5 is one frequently-used self-paced regularization term, and item 6 balances the average weights of positive samples and negative samples. Notably, items 7 and 9 make up the consistency regularization term that expects the model to recognize different data enhancement versions of the same image correctly.

**Figure 1:**
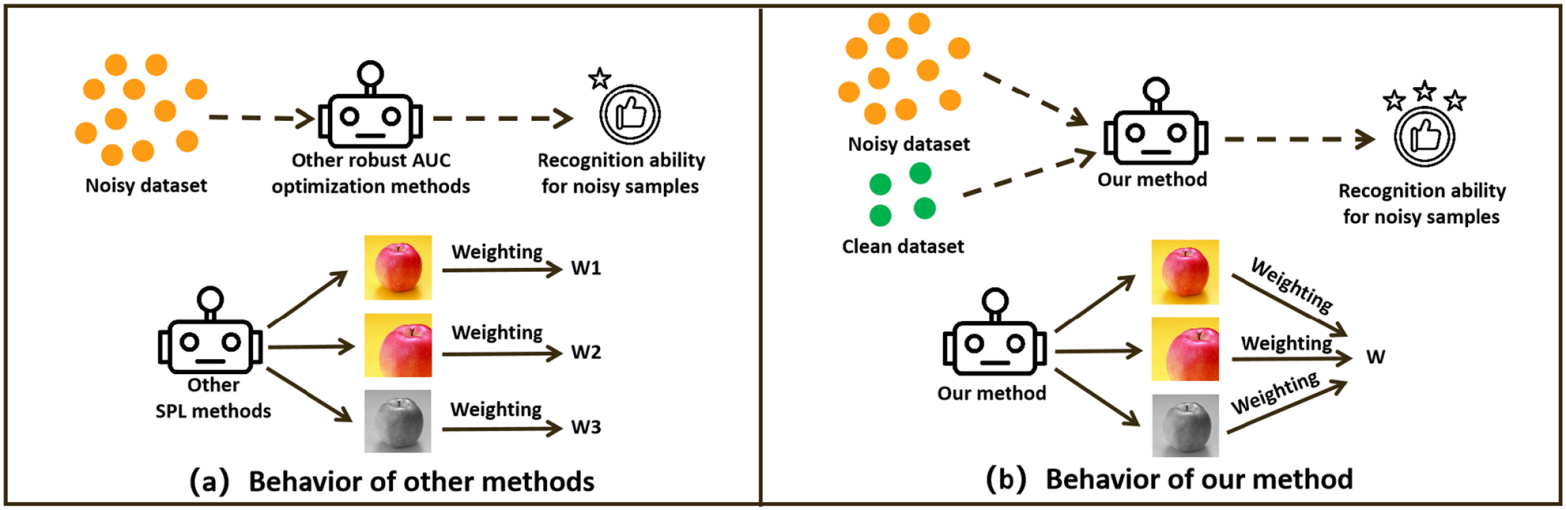
Advantages of our RAUCO algorithm.

We provide Fig. 1 to explain our advantages figuratively, and Fig. 1a,b show the behavior of other algorithms and our RAUCO algorithm respectively. As shown in Fig. 1a, existing AUC optimization algorithms only consider the use of the noisy dataset, and existing SPL methods suffer from the problem that they would assign inconsistent weights to different data enhancement versions of the same image. However, as shown in Fig. 1b, our RAUCO algorithm emphasizes the importance of the small set of clean samples. In this case, our RAUCO algorithm has a more vital ability to identify and eliminate noisy samples. Moreover, by introducing the consistency regularization term, our RAUCO algorithm tries to assign the consistent weight to different data enhancement version of the same image.

### RAUCO algorithm

In this subsection, we give a detailed explanation of our RAUCO algorithm.

*Sampling stochastically.* Considering that we utilize both the small set of clean data and the large-scale noisy data, let $\widetilde{\mathscr {S}}=\{(\widetilde{\mathbf{x}}_i,\widetilde{y}_i)\}_{i=1}^m$ and $\mathscr {S}=\{(\mathbf{x}_i,y_i)\}_{i=1}^n$ be the clean dataset and the noisy dataset respectively. For efficiency, we utilize the stochastic gradient instead of the full gradient. Specifically, in *t*-iteration, we stochastically sample a clean mini-batch $\widetilde{\mathscr {B}}^t= \{(\widetilde{\mathbf{x}}_i,\widetilde{y}_i)\}_{i=1}^{m_{\widetilde{\mathscr {B}}^t}}$ with $m_{\widetilde{B}^t}^+$ positive samples and $m_{\widetilde{B}^t}^-$ negative samples, and stochastically sample a noisy mini-batch ${\mathscr {B}}^t= \{({\mathbf{x}}_i,{y}_i)\}_{i=1}^{n_{{\mathscr {B}}^t}}$ with $n_{{B}^t}^+$ positive samples and $n_{{B}^t}^-$ negative samples.

*Calculating stochastic gradients with respect to model parameters.*. With fixed sample weights $\mathbf{w}$, our objective function, *i.e.*, Eq. (4), degenerates into one particular AUC optimization objective. In this case, the stochastic gradient with respect to model parameters $\theta$ is formulated as:

5 $$\begin{aligned} \mathscr {G}_{\theta }(\theta )=&\underbrace{2 \tau | \theta | }_{{\mathbf {1}}} \underbrace{+ 2 \gamma | \theta - \widetilde{\theta }^* |}_{{\mathbf {2}}} \underbrace{ + \sum _{i=1}^{m_{\widetilde{\mathscr {B}}^t}^+} \sum _{j=1}^{m_{\widetilde{\mathscr {B}}^t}^-} \frac{ 1 }{m_{\widetilde{\mathscr {B}}^t}^+ m_{\widetilde{\mathscr {B}}^t}^-} \frac{\partial \xi (\widetilde{\mathbf{x}}^+_i, \widetilde{\mathbf{x}}^-_j)}{ \partial \theta } + \sum _{i=1}^{n_{\mathscr {B}^t}^+} \sum _{j=1}^{n_{\mathscr {B}^t}^-} \frac{ w^+_i w^-_j }{n_{\mathscr {B}^t}^+ n_{\mathscr {B}^t}^-} \frac{\partial \xi ({\mathbf{x}}^+_i,{\mathbf{x}}^-_j) }{ \partial \theta }}_{{\mathbf {3}}} \nonumber \\&\underbrace{ + \rho \sum _{i=1}^{n_{\mathscr {B}^t}} \frac{1}{n_{\mathscr {B}^t}} \frac{ \partial \zeta (\mathbf{x}_i) }{\partial \theta }+ \rho \sum _{j=1}^{m_{\widetilde{\mathscr {B}}^t}} \frac{ 1}{m_{\widetilde{\mathscr {B}}^t}} \frac{ \partial \zeta (\widetilde{\mathbf{x}}_j) }{\partial \theta }}_{{\mathbf {4}}}. \end{aligned}$$

The stochastic gradient $\mathscr {G}_{\theta }$ with respect to $\theta$ mainly consists of four parts. Say concretely, item 1 is related to the model regularization term used to avoid overfitting, and item 2 emphasizes the importance of clean samples and expects that the difference between the current model and the model obtained from training clean samples would be insignificant. Also, item 3 is generated from the AUC loss of clean samples and noisy samples. Item 4 attempts to make the model predictions of different data enhancement versions of the same image as same as possible, so that our algorithm can assign reasonable weights to different data enhancement versions.

*Calculating stochastic gradients with respect to sample weights*. If we fix the model parameters $\theta$ in our objective function, *i.e.*, Eq. (4), the stochastic gradients with respect to weights $w_i^+, w_j^-$ of the positive sample and the negative sample are formulated as:

6 $$\begin{aligned} \begin{aligned} \mathscr {G}^+_w(w_i^+)&= \frac{1}{n^+} \Bigg ( \sum _{j=1}^{ n_{\mathscr {B}^t}^-} \frac{ w_j^- \xi (\mathbf{x}^+_i,\mathbf{x}^-_j) }{ n_{\mathscr {B}^t}^-} - \lambda + 2 \mu \bigg ( \sum _{i=1}^{n^+} \frac{w_i^+}{n^+} - \sum _{j=1}^{n^-}\frac{w_j^-}{n^-} \bigg ) \Bigg ), \ \ \mathscr {G}^-_w(w_j^-)\\&= \frac{1}{n^-} \Bigg ( \sum _{i=1}^{ n_{\mathscr {B}^t}^+} \frac{ w_i^+ \xi (\mathbf{x}^+_i,\mathbf{x}^-_j) }{ n_{\mathscr {B}^t}^+} - \lambda + 2 \mu \bigg ( \sum _{j=1}^{n^-}\frac{w_j^-}{n^-} - \sum _{i=1}^{n^+} \frac{w_i^+}{n^+} \bigg ) \Bigg ). \end{aligned} \end{aligned}$$

For noisy samples with significant losses, the stochastic gradients of their weights can also be significant. Consequently, their weights will gradually decrease until they reach zero, effectively removing any negative impact on the model.

**Algorithm 1:**
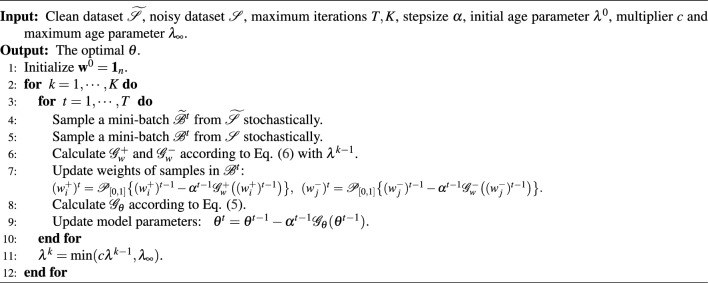
RAUCO Algorithm

*Summarizing the RAUCO algorithm*. Above all, we summarize our RAUCO algorithm in Algorithm 1, where $\mathscr {P}_{[0,1]} \{ \cdot \}$ means the projection operation to [0, 1]. Specifically, we first sample the clean mini-batch $\widetilde{\mathscr {B}}^t$ and the noisy mini-batch ${\mathscr {B}}^t$ stochastically *i.e.*, lines 4 and 5. Based on these samples, we employ the stochastic gradients with respect to model parameters $\theta$ and sample weights $\mathbf{w}$ to conduct model updates, *i.e.*, lines 6 - 9. Following the setting of SPL^22^, we gradually increase the age parameter $\lambda$ to allow more samples to join the training, *i.e.*, line 11.

*Challenges and innovations*. The BSPAUC algorithm^16^ introduces the SPL technology into the AUC optimization problem and is most relevant to our RAUCO algorithm. The main differences between these two algorithms are as follows:

1. The BSPAUC algorithm faces a challenge where a model, misled by noisy samples during stochastic optimization, ultimately results in unsatisfactory performance. Conversely, by incorporating items 2, 3 and 8 in Eq. (4), our RAUCO algorithm strategically employs clean samples to steer the training process. This approach guarantees that the evolving model remains closely aligned with the ideal model, thereby enhancing its accuracy and reliability.
2. Same as other SPL methods, the BSPAUC algorithm suffers from the negative impact of the data enhancement technology, *i.e.*, the BSPAUC algorithm would assign inconsistent weights to different data enhancement versions of the same image. However, our RAUCO algorithm solves this problem by introducing the consistency regularization term, *i.e.*, items 7 and 9 in Eq. (4).
3. The BSPAUC algorithm optimizes the objective function by the traditional alternative optimization strategy, which alternatively minimizes the two critical sub-problems with respect to sample weights and model parameters. In this case, the time complexity of the BSPAUC algorithm is $O(T n^2)$ because it needs to calculate an enormous matrix with the shape of $n^2$ in each iteration. To avoid this situation, our RAUCO algorithm alternatively optimizes sample weights and model parameters with the stochastic gradients. Then, the time complexity of our RAUCO algorithm is $O(T n^2_{\mathscr {B}})$. Because of $n \gg n_{\mathscr {B}}$, our RAUCO algorithm is more efficient. Importantly, we prove the convergence of our RAUCO algorithm in the next section.

## Theoretical analysis

In this section, we prove the convergence of our RAUCO algorithm.

We first introduce some definitions. Let $G_{\theta }^t$ and $G_{\mathbf{w}}^t$ be the stochastic gradients with respect to $\theta$ and $\mathbf{w}$ generated by Algorithm 1 in t-iteration. Importantly, for $G_{\mathbf{w}}^t \in \mathbb{R}^n$, if the stochastic gradient with respect to $w_i, i \in [n]$ is not calculated by Algorithm 1 in t-iteration, the *i*-th element of $G_{\mathbf{w}}^t$ is 0. For the vector $\mathbf{e}^t \in \{0,1\}^n$, if i-th element of $G_{\mathbf{w}}^t$ is 0, $\mathbf{e}_i^t=0$; otherwise, $\mathbf{e}_i^t=1$. Also, for the operator $\otimes$, if $A \otimes B = C$ and $A, B, C \in \mathbb{R}^n$, we have $A_i * B_i = C_i, i \in [n]$. Finally, $\mathscr {P}_S( \cdot )$ is the projection operation to the set *S*.

Then, we provide some necessary assumptions and the definition of the projected gradient.

### Assumption 1

(*Lipschitz Smooth*) For our objective function $\mathscr {L}_{\lambda }(\theta ,\mathbf{w})$, the sub-problems with respect to $\theta$ and $\mathbf{w}$, *i.e.*, $\mathscr {L}_{\lambda }(\theta ;\mathbf{w})$ and $\mathscr {L}_{\lambda }(\mathbf{w};\theta )$, are both Lipschitz smooth with the maximum Lipschitz constant *L*, *i.e.*, $\forall \theta , \theta '$, and $\forall \mathbf{w}, \mathbf{w}' \in [0,1]^n$, we have:

7 $$\begin{aligned} || \mathscr {L}_{\lambda }(\theta ; \mathbf{w}) - \mathscr {L}_{\lambda }(\theta ' ; \mathbf{w}) || \le L ||\theta - \theta '||, \ \ || \mathscr {L}_{\lambda }(\mathbf{w}; \theta ) - \mathscr {L}_{\lambda }(\mathbf{w}' ; \theta ) || \le L ||\mathbf{w}- \mathbf{w}'||. \end{aligned}$$

### Assumption 2

For $\forall t \in [T]$, we have

8 $$\begin{aligned} \begin{aligned} \mathbb{E} \big [ || G_{\mathbf{w}}^t - \mathbf{e}^t \otimes \nabla _{\mathbf{w}} \mathscr {L}_{\lambda }(\mathbf{w}^t;\theta ^t) ||_2^2 \big ] \le (\sigma ^t_{\mathbf{w}})^2, \ \ \mathbb{E} \big [ || G_{\theta }^t - \nabla _{\theta } \mathscr {L}_{\lambda }(\theta ^t;\mathbf{w}^{t+1}) ||_2^2 \big ] \le (\sigma ^t_{\theta })^2, \end{aligned} \end{aligned}$$

where $\sigma ^t_{\mathbf{w}}>0$, $\sigma ^t_{\theta }>0$ are some constants and we define $\sigma ^t=\max \{\sigma ^t_{\mathbf{w}}, \sigma ^t_{\theta }\}$.

### Definition 1

(*Projected gradient*)^28^ Let *S* be a closed convex set with dimension *N*, and the projected gradient is defined as:

9 $$\begin{aligned} \mathscr {K}(\mathbf{w},\mathbf{g},\alpha )=\frac{1}{\alpha }(\mathbf{w}-\mathscr {P}_\mathscr {S}(\mathbf{w}-\alpha \mathbf{g} )) \end{aligned}$$

where $$\mathbf{w}\in S$$, $$\mathbf{g} \in \mathbb{R}^N$$ and $$\alpha \in \mathbb{R}^+$$.

Assumptions 1 and 2 are common assumptions in stochastic optimization. Assumption 1 provides the guarantee of the Lipschitz smoothness, and Assumption 2 bounds the difference between the stochastic gradient and the full gradient.

Finally, our theoretical result is as follows and the specific proof process can be found in Appendix.

### Theorem 1

When Assumptions 1 and 2 hold, $\lambda$ reaches its maximum value $\lambda _{\infty }$ and the stepsizes $\{\alpha ^t\}_{t=1}^{\infty }$ satisfy

10 $$\begin{aligned} 0< \alpha ^{t+1} \le \alpha ^{t}< \frac{2}{L}, \quad \sum _{t=1}^{\infty } \alpha ^t = + \infty , \quad \sum _{t=1}^{\infty } \alpha ^t (\sigma ^t)^2 < \infty , \end{aligned}$$

then there exists an index sub-sequence $\mathscr {M}$ in Algorithm 1 such that

11 $$\begin{aligned} \lim \limits _{\begin{array}{c} t \rightarrow \infty \\ t\in \mathscr {M} \end{array} }\mathbb{E} ||\frac{(\theta ^{t+1},\mathbf{w}^{t+1})-(\theta ^{t},\mathbf{w}^{t}) }{\alpha ^t} ||_2^2 =0. \end{aligned}$$

The above theorem shows that Algorithm 1 approaches a stationary point of our objective function. It indicates that our algorithm can obtain a satisfactory solution theoretically.

## Experiments

In this section, we describe the experimental setup and then provide our experimental results and discussion.

### Experimental setup

*Compared algorithms*. We compare our RAUCO algorithm with the standard AUC optimization algorithms and the noise-robust AUC optimization algorithms. Specifically, the compared algorithms are summarized as follows:

1. DSAM^10^ is a standard AUC optimization algorithm that optimizes the model based on the doubly stochastic gradient with respect to the doubly stochastic sampling.
2. TSAM^11^ is a standard AUC optimization algorithm that optimizes the model based on the triply stochastic gradient with respect to the doubly stochastic sampling and the stochastic feature.
3. RDAM^14^ is a noise-robust AUC optimization algorithm that utilizes a more robust AUC margin loss than the commonly used AUC square loss.
4. BSPAUC^16^ is a noise-robust AUC optimization algorithm that deals with noisy samples using the SPL technology.

*Datasets* The experiments are conducted on the standard datasets: MNIST^29^, CIFAR10^30^ and SVHN^31^. For the setting of the two-class AUC optimization problem, we select two similar classes from each dataset and then set their sample ratio to 10 : 1. For MNIST and SVHN, we conduct the experiments on “0 versus 8” and “6 versus 9”. For CIFAR10, we conduct the experiments on “automobile versus truck” and “deer versus horse”. For each dataset, we select 50 positive samples and 50 negative samples to form the small set of available clean samples. Moreover, we artificially construct noisy datasets by flipping the labels of clean samples^17,32^.

**Table 2:** Mean AUC results with the corresponding standard deviation on original benchmark datasets.

	MNIST		CIFAR10		SVHN	
	0 versus 8	6 versus 9	automobile versus truck	Deer versus horse	0 versus 8	6 versus 9
TSAM	0.994 ± 0.002	0.991 ± 0.002	0.885 ± 0.002	0.864 ± 0.001	0.938 ± 0.004	0.928 ± 0.004
DSAM	0.992 ± 0.001	0.995 ± 0.002	0.881 ± 0.004	0.871 ± 0.003	0.934 ± 0.005	0.936 ± 0.004
RDAM	0.992 ± 0.002	0.993 ± 0.002	0.884 ± 0.003	0.852 ± 0.003	0.927 ± 0.004	0.932 ± 0.004
BSPAUC	0.993 ± 0.001	0.995 ± 0.001	0.894 ± 0.003	0.877 ± 0.002	0.942 ± 0.002	0.956 ± 0.002
RAUCO	**0.999 ± 0.001**	**0.999 ± 0.001**	**0.907 ± 0.003**	**0.892 ± 0.003**	**0.958 ± 0.003**	**0.974 ± 0.002**
Win/Tie/Loss	4/0/0	3/1/0	4/0/0	4/0/0	4/0/0	4/0/0

Significant values are in bold.

**Table 3:** Mean AUC results with the corresponding standard deviation on noisy benchmark datasets.

	MNIST: 0 versus 8			MNIST: 6 versus 9		
	NR: 10%	NR: 20%	NR: 30%	NR: 10%	NR: 20%	NR: 30%
TSAM	0.961 ± 0.003	0.938 ± 0.003	0.904 ± 0.003	0.961 ± 0.002	0.942 ± 0.002	0.916 ± 0.003
DSAM	0.964 ± 0.004	0.937 ± 0.005	0.908 ± 0.002	0.954 ± 0.003	0.935 ± 0.002	0.903 ± 0.001
RDAM	0.971 ± 0.003	0.955 ± 0.003	0.933 ± 0.002	0.974 ± 0.002	0.942 ± 0.002	0.916 ± 0.002
BSPAUC	0.975 ± 0.003	0.960 ± 0.006	0.940 ± 0.003	0.977 ± 0.003	0.948 ± 0.001	0.923 ± 0.002
RAUCO	**0.991 ± 0.002**	**0.975 ± 0.004**	**0.962 ± 0.003**	**0.986 ± 0.002**	**0.967 ± 0.003**	**0.954 ± 0.002**
Win/Tie/Loss	4/0/0	4/0/0	4/0/0	4/0/0	4/0/0	4/0/0

	CIFAR10: automobile versus truck			CIFAR10: deer versus horse		
	NR: 10%	NR: 20%	NR: 30%	NR: 10%	NR: 20%	NR: 30%
TSAM	0.843 ± 0.001	0.808 ± 0.002	0.765 ± 0.003	0.829 ± 0.002	0.794 ± 0.002	0.762 ± 0.002
DSAM	0.836 ± 0.003	0.795 ± 0.002	0.757 ± 0.003	0.823 ± 0.001	0.782 ± 0.001	0.744 ± 0.001
RDAM	0.849 ± 0.001	0.803 ± 0.002	0.772 ± 0.004	0.826 ± 0.001	0.794 ± 0.002	0.767 ± 0.002
BSPAUC	0.858 ± 0.003	0.819 ± 0.004	0.786 ± 0.002	0.842 ± 0.001	0.808 ± 0.001	0.773 ± 0.002
RAUCO	**0.875 ± 0.002**	**0.843 ± 0.002**	**0.820 ± 0.001**	**0.864 ± 0.002**	**0.841 ± 0.002**	**0.817 ± 0.003**
Win/Tie/Loss	4/0/0	4/0/0	4/0/0	4/0/0	4/0/0	4/0/0

	SVHN: 0 versus 8			SVHN: 6 versus 9		
	NR: 10%	NR: 20%	NR: 30%	NR: 10%	NR: 20%	NR: 30%
TSAM	0.914 ± 0.004	0.878 ± 0.005	0.836 ± 0.003	0.894 ± 0.006	0.863 ± 0.004	0.841 ± 0.002
DSAM	0.917 ± 0.003	0.883 ± 0.006	0.844 ± 0.003	0.902 ± 0.006	0.874 ± 0.002	0.835 ± 0.002
RDAM	0.909 ± 0.006	0.888 ± 0.003	0.852 ± 0.003	0.909 ± 0.004	0.885 ± 0.005	0.851 ± 0.002
BSPAUC	0.919 ± 0.005	0.893 ± 0.004	0.855 ± 0.003	0.930 ± 0.002	0.904 ± 0.003	0.863 ± 0.004
RAUCO	**0.942 ± 0.005**	**0.924 ± 0.003**	**0.895 ± 0.003**	**0.960 ± 0.001**	**0.936 ± 0.003**	**0.901 ± 0.003**
Win/Tie/Loss	4/0/0	4/0/0	4/0/0	4/0/0	4/0/0	4/0/0

Significant values are in bold.

**Figure 2:**
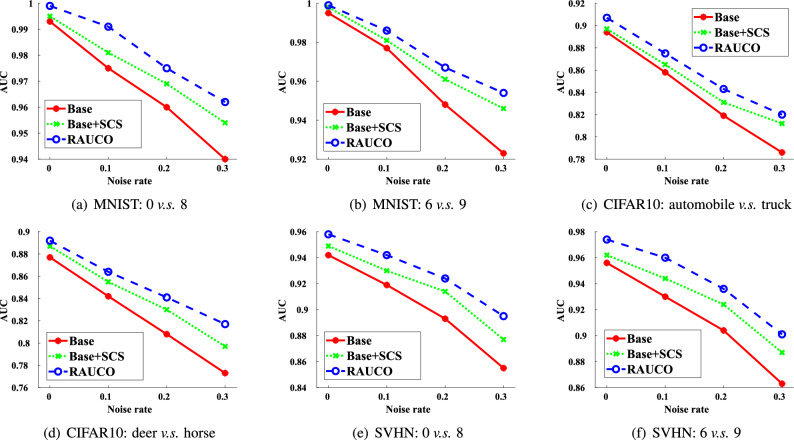
Results of ablation experiments.

**Figure 3:**
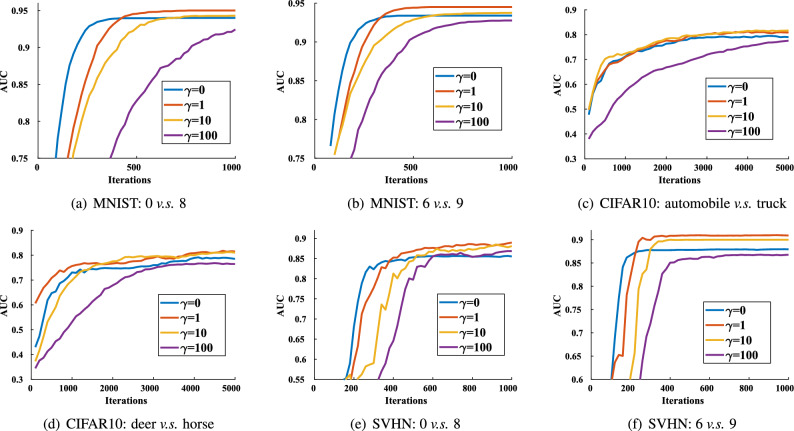
Experimental results with different values of the hyperparameter $\gamma$ on datasets with $30\%$ noisy samples.

*Design of experiments* We first show the experimental results on original datasets in Table 2. For the noise robustness, we show the experimental results with different noise ratios (from 10 to 30%) in Table 3. Importantly, as shown in Fig. 2, we conduct the ablation experiments for the effectiveness of each component of our RAUCO algorithm. Also, we pay attention to the effect of the hyperparameter $\gamma$ and show experimental results with different values of $\gamma$ on datasets with $30\%$ noisy samples in Fig. 3.

*Experimental details* All experiments were carried out on a computer equipped with four NVIDIA RTX A6000 GPUs, and the reported results represent the average of 10 trials. All algorithms were implemented using Python, employing identical network structures and optimized through Stochastic Gradient Descent^33^. In the case of the BSPAUC algorithm^16^, we fine-tuned the parameter $\lambda _{\infty }$ within the range of [0, 2], and we set $\mu$ to the same value as $\lambda _{\infty }$. For the TSAM algorithm^11^, we varied the number of random Fourier features within the interval [500, 4000] in increments of 500. Regarding the balanced self-paced component of our RAUCO algorithm, we applied the same settings as those used for the BSPAUC algorithm^16^. Additionally, we conducted a parameter search for $\gamma$ and $\rho$ within the range of $5^{ \{-3,-2,-1,0,1,2\} }$ using a 10-fold cross-validation approach^34^.

*Significance test* Based on the experimental results, we also perform the significance test. Specifically, we show Win/Tie/Loss counts of our RAUCO algorithm against other algorithms with the t-test at a significance level of 0.05. The Win/Loss count represents the number of algorithms whose performance is significantly lower/higher than that of our RAUCO algorithm, and the Tie count represents the number of algorithms whose performance is not significantly different from that of our RAUCO algorithm.

### Results and discussion

Table 2 presents the AUC performance on standard datasets. Thanks to the supervision of clean samples and the consistency regularization term, our RAUCO algorithm can make better use of the advantages of the SPL technology, which could avoid the model getting stuck into the bad local optimal solution and improve the model performance. Thus, our RAUCO algorithm obtains better generalization ability than the BSPAUC algorithm that is the base model of our RAUCO algorithm. Meanwhile, compared with other state-of-the-art AUC optimization algorithms, our RAUCO also obtains better performance.

Table 3 shows AUC performance on noisy datasets with different noise ratios (from 10 to 30%). These results show that our RAUCO obviously achieves better performance than non-robust AUC optimization algorithms. Compared with robust AUC optimization algorithms, our RAUCO also has advantages. Specifically, while the RDAM algorithm attempts to reduce the influence of noisy samples, it is still sensitive to large noise ratios. Meanwhile, the BSPAUC algorithm does not make good use of clean samples and does not solve the unreasonable weighting problem of the data enhancement version. Thus, the BSPAUC algorithm gets unsatisfactory performance.

Figure 2 shows the AUC performance of the ablation experiments on datasets with different noise ratios (from 0 to 30%), where the base model represents the BSPAUC algorithm and the SCS means the strategy of training under the supervision of clean samples. With the SCS strategy, the performance of the base model is improved regardless of the noise ratios. However, this algorithm still faces the unreasonable weighting problem of the data enhancement version. Benefiting from the consistency regularization term, our RAUCO algorithm thus achieves the best performance.

Figure 3 shows experimental results with different values of $\gamma$ on datasets with $30\%$ noisy samples. When $\gamma$ is 0, our RAUCO algorithm only trains clean samples simply, so it does not produce a satisfactory performance. With the increase of $\gamma$, our RAUCO algorithm expects that the difference between the current model and the model obtained from training clean samples would be insignificant. In this case, our RAUCO algorithm employs clean samples to guide the training of the current model and thus has a more vital ability to identify and eliminate noisy samples. However, when $\gamma$ is too large, the current model will be limited to the vicinity of the model obtained from training clean samples, resulting in reduced generalization.

## Conclusion

In this paper, we propose a robust AUC optimization (RAUCO) algorithm to make full use of both clean data and noisy data. Say concretely, benefiting from the SPL technology, our RAUCO algorithm can exclude noisy samples from the training under the supervision of clean samples. Moreover, considering the negative impact of the data enhancement technology on SPL, we innovatively introduce the consistency regularization term. Compared with traditional SPL methods that need to alternately solve the two critical sub-problems with respect to sample weights and model parameters, our RAUCO algorithm is more efficient by updating sample weights and model parameters alternately with the stochastic gradient method. Theoretical results show the convergence of our RAUCO algorithm, and experimental results demonstrate that our RAUCO algorithm holds better robustness than existing algorithms.

### Supplementary Information

Supplementary Information.

## Data Availability

The datasets used and/or analyzed during the current study available from the corresponding author on reasonable request.
