# Supplementary material for: Robust AUC optimization under the supervision of clean data
Source: Sci Rep. 2024 Jul 19;14:16693. doi: 10.1038/s41598-024-66788-2 (PMC11271567; doi:10.1038/s41598-024-66788-2)
Supplement: Supplementary file 1 — Supplementary Information. [file 41598_2024_66788_MOESM1_ESM.pdf]

Supplementary Material for "Robust AUC Optimization under the Supervision of Clean Data"

Chenkang Zhang[1,*], Haobing Tian[1], Lang Zhang[1], and Pengju Jiao[1]

[1]China Mobile (Suzhou) Software Technology Company Limited, 215163 Suzhou, China
[*]zhangchenkang@cmss.chinamobile.com

Proof of Theorem 1

In this section, we prove the convergence of our RAUCO algorithm.

We first introduce some definitions. Let G^t_θ and $G^t_\mathbf{w}$ be the stochastic gradients with respect to θ and $\mathbf{w}$ generated by Algorithm 1 in t-iteration. Importantly, for $G^t_\mathbf{w} \in \mathbb{R}^n$, if the stochastic gradient with respect to $w_i, i \in [n]$ is not calculated by Algorithm 1 in t-iteration, the i-th element of $G^t_\mathbf{w}$ is 0. For the vector $\mathbf{e}^t \in \{0,1\}^n$, if i-th element of $G^t_\mathbf{w}$ is 0, $\mathbf{e}^t_i = 0$; otherwise, $\mathbf{e}^t_i = 1$. Also, for the operator $\otimes$, if $A \otimes B = C$ and $A, B, C \in \mathbb{R}^n$, we have $A_i * B_i = C_i, i \in [n]$. Finally, $\mathscr{P}_S(\cdot)$ is the projection operation to the set S.

Then, we provide some necessary assumptions and the definition of the projected gradient.

Assumption 1 (Lipschitz Smooth)**.** *For our objective function $\mathscr{L}_\lambda(\theta, \mathbf{w})$, the sub-problems with respect to θ and $\mathbf{w}$,* i.e., *$\mathscr{L}_\lambda(\theta; \mathbf{w})$ and $\mathscr{L}_\lambda(\mathbf{w}; \theta)$, are both Lipschitz smooth with the maximum Lipschitz constant L,* i.e., *$\forall \theta, \theta'$, and $\forall \mathbf{w}, \mathbf{w}' \in [0,1]^n$, we have:*

$$||\mathscr{L}_\lambda(\theta; \mathbf{w}) - \mathscr{L}_\lambda(\theta'; \mathbf{w})|| \leq L||\theta - \theta'||, \ \ ||\mathscr{L}_\lambda(\mathbf{w}; \theta) - \mathscr{L}_\lambda(\mathbf{w}'; \theta)|| \leq L||\mathbf{w} - \mathbf{w}'||. \tag{1}$$

Assumption 2. *For $\forall t \in [T]$, we have*

$$\mathbb{E}[||G^t_\mathbf{w} - \mathbf{e}^t \otimes \nabla_\mathbf{w} \mathscr{L}_\lambda(\mathbf{w}^t; \theta^t)||^2_2] \leq (\sigma^t_\mathbf{w})^2, \ \ \mathbb{E}[||G^t_\theta - \nabla_\theta \mathscr{L}_\lambda(\theta^t; \mathbf{w}^{t+1})||^2_2] \leq (\sigma^t_\theta)^2, \tag{2}$$

where $\sigma^t_\mathbf{w} > 0$, $\sigma^t_\theta > 0$ are some constants and we define $\sigma^t = \max\{\sigma^t_\mathbf{w}, \sigma^t_\theta\}$.

Definition 1 (Projected Gradient)**.** [1] *Let S be a closed convex set with dimension N, and the projected gradient is defined as:*

$$\mathscr{K}(\mathbf{w}, \mathbf{g}, \alpha) = \frac{1}{\alpha}(\mathbf{w} - \mathscr{P}_\mathscr{S}(\mathbf{w} - \alpha \mathbf{g})) \tag{3}$$

where $\mathbf{w} \in S$, $\mathbf{g} \in \mathbb{R}^N$ and $\alpha \in \mathbb{R}^+$.

Assumptions 1 and 2 are common assumptions in stochastic optimization. Assumption 1 provides the guarantee of the Lipschitz smoothness, and Assumption 2 bounds the difference between the stochastic gradient and the full gradient.

Finally, our theoretical result is as follows.

Theorem 1. *When Assumptions 1 and 2 hold, λ reaches its maximum value λ_∞ and the stepsizes $\{\alpha^t\}^\infty_{t=1}$ satisfy*

$$0 < \alpha^{t+1} \leq \alpha^t < \frac{2}{L}, \ \ \sum_{t=1}^{\infty} \alpha^t = +\infty, \ \ \sum_{t=1}^{\infty} \alpha^t (\sigma^t)^2 < \infty, \tag{4}$$

then there exists an index sub-sequence $\mathscr{M}$ in Algorithm 1 such that

$$\lim_{\substack{t \to \infty \\ t \in \mathscr{M}}} \mathbb{E}||\frac{(\theta^{t+1}, \mathbf{w}^{t+1}) - (\theta^t, \mathbf{w}^t)}{\alpha^t}||^2_2 = 0. \tag{5}$$

The above theorem shows that Algorithm 1 approaches a stationary point of our objective function. It indicates that our algorithm can obtain a satisfactory solution theoretically.

Before proving our theoretical results (*i.e.*, Theorem 1), we introduce the following lemmas.

Lemma 1. [1] *Let S be a closed convex set with dimension N, for any $\mathbf{w} \in S$, $\mathbf{g} \in \mathbb{R}^N$ and $\alpha > 0$, we have*

$$\langle \mathbf{g}, \mathscr{K}(\mathbf{w}, \mathbf{g}, \alpha) \rangle \geq ||\mathscr{K}(\mathbf{w}, \mathbf{g}, \alpha)||^2_2. \tag{6}$$

Lemma 2. [1] *Let S be a closed convex set with dimension N, for any $\mathbf{w} \in S$, $\alpha > 0$ and $\mathbf{g}_1, \mathbf{g}_2 \in \mathbb{R}^N$, we have*

$$||\mathscr{K}(\mathbf{w}, \mathbf{g}_1, \alpha) - \mathscr{K}(\mathbf{w}, \mathbf{g}_2, \alpha)||_2 \le ||\mathbf{g}_1 - \mathbf{g}_2||_2. \tag{7}$$

Lemma 3. [2] *For two nonnegative scalar sequences $\{a^t\}_{t=1}^{\infty}$ and $\{b^t\}_{t=1}^{\infty}$, if $\sum_{t=1}^{\infty} a^t = +\infty$ and $\sum_{t=1}^{\infty} a^t b^t < +\infty$, then*

$$\lim_{t \to +\infty} \inf b^t = 0.$$

Finally, the proof of Theorem 1 is as follows.

Proof. Here, we define some concepts of the gradient with respect to $\mathbf{w}$:

$$K_{\mathbf{w}}^t = \mathscr{K}(\mathbf{w}^t, \mathbf{e}^t \otimes \nabla_{\mathbf{w}} \mathscr{L}_\lambda(\mathbf{w}^t; \theta^t), \alpha^t), \;\; k_{\mathbf{w}}^t = \mathscr{K}(\mathbf{w}^t, G_{\mathbf{w}}^t, \alpha^t), \;\; \delta_{\mathbf{w}}^t = G_{\mathbf{w}}^t - \mathbf{e}^t \otimes \nabla_{\mathbf{w}} \mathscr{L}_\lambda(\mathbf{w}^t; \theta^t). \tag{8}$$

Next are some similar concepts of the gradient with respect to θ:

$$K_\theta^t = \nabla_\theta \mathscr{L}_\lambda(\theta^t; \mathbf{w}^{t+1}), \;\; k_\theta^t = G_\theta^t, \;\; \delta_\theta^t = G_\theta^t - \nabla_\theta \mathscr{L}_\lambda(\theta^t; \mathbf{w}^{t+1}). \tag{9}$$

According to the optimization process, we have

$$\mathscr{L}_\lambda(\theta^{t+1}, \mathbf{w}^{t+1}) - \mathscr{L}_\lambda(\theta^t, \mathbf{w}^t) = \mathscr{L}_\lambda(\theta^{t+1}, \mathbf{w}^{t+1}) - \mathscr{L}_\lambda(\theta^t, \mathbf{w}^{t+1}) + \mathscr{L}_\lambda(\theta^t, \mathbf{w}^{t+1}) - \mathscr{L}_\lambda(\theta^t, \mathbf{w}^t).$$

Due to that the sub-problem $\mathscr{L}_\lambda(\mathbf{w}; \theta)$ with respect to $\mathbf{w}$ is Lipschitz smooth with the Lipschitz constant L, we have

$$\begin{aligned}
&\mathscr{L}_\lambda(\mathbf{w}^{t+1}; \theta^t) - \mathscr{L}_\lambda(\mathbf{w}^t; \theta^t) \;\le\; \langle \nabla_{\mathbf{w}} \mathscr{L}_\lambda(\mathbf{w}^t; \theta^t), \mathbf{w}^{t+1} - \mathbf{w}^t \rangle + \frac{L}{2} ||\mathbf{w}^{t+1} - \mathbf{w}^t||_2^2 \\
&= -\alpha^t \langle \nabla_{\mathbf{w}} \mathscr{L}_\lambda(\mathbf{w}^t; \theta^t), k_{\mathbf{w}}^t \rangle + \frac{L(\alpha^t)^2}{2} ||k_{\mathbf{w}}^t||_2^2 \;=\; -\alpha^t \langle \mathbf{e}^t \otimes \nabla_{\mathbf{w}} \mathscr{L}_\lambda(\mathbf{w}^t; \theta^t), k_{\mathbf{w}}^t \rangle + \frac{L(\alpha^t)^2}{2} ||k_{\mathbf{w}}^t||_2^2 \\
&= -\alpha^t \langle G_{\mathbf{w}}^t, k_{\mathbf{w}}^t \rangle + \frac{L(\alpha^t)^2}{2} ||k_{\mathbf{w}}^t||_2^2 + \alpha^t \langle G_{\mathbf{w}}^t - \mathbf{e}^t \otimes \nabla_{\mathbf{w}} \mathscr{L}_\lambda(\mathbf{w}^t; \theta^t), k_{\mathbf{w}}^t \rangle \;=\; -\alpha^t \langle G_{\mathbf{w}}^t, k_{\mathbf{w}}^t \rangle + \frac{L(\alpha^t)^2}{2} ||k_{\mathbf{w}}^t||_2^2 + \alpha^t \langle \delta_{\mathbf{w}}^t, k_{\mathbf{w}}^t \rangle
\end{aligned}$$

Then, by Lemma 1 with $\mathbf{w} = \mathbf{w}^t, \mathbf{g} = G_{\mathbf{w}}^t$ and $\alpha = \alpha^t$, we obtain

$$\begin{aligned}
&\mathscr{L}_\lambda(\mathbf{w}^{t+1}; \theta^t) - \mathscr{L}_\lambda(\mathbf{w}^t; \theta^t) \;\le\; -\alpha^t ||k_{\mathbf{w}}^t||_2^2 + \frac{L(\alpha^t)^2}{2} ||k_{\mathbf{w}}^t||_2^2 + \alpha^t \langle \delta_{\mathbf{w}}^t, k_{\mathbf{w}}^t \rangle \\
&= (-\alpha^t + \frac{L(\alpha^t)^2}{2}) ||k_{\mathbf{w}}^t||_2^2 + \alpha^t \langle \delta_{\mathbf{w}}^t, K_{\mathbf{w}}^t \rangle + \alpha^t \langle \delta_{\mathbf{w}}^t, k_{\mathbf{w}}^t - K_{\mathbf{w}}^t \rangle \;\le\; (-\alpha^t + \frac{L(\alpha^t)^2}{2}) ||k_{\mathbf{w}}^t||_2^2 + \alpha^t \langle \delta_{\mathbf{w}}^t, K_{\mathbf{w}}^t \rangle + \alpha^t ||\delta_{\mathbf{w}}^t||_2 ||k_{\mathbf{w}}^t - K_{\mathbf{w}}^t||_2 \\
&\le (-\alpha^t + \frac{L(\alpha^t)^2}{2}) ||k_{\mathbf{w}}^t||_2^2 + \alpha^t \langle \delta_{\mathbf{w}}^t, K_{\mathbf{w}}^t \rangle + \alpha^t ||\delta_{\mathbf{w}}^t||_2 ||\delta_{\mathbf{w}}^t||_2 \;=\; (-\alpha^t + \frac{L(\alpha^t)^2}{2}) ||k_{\mathbf{w}}^t||_2^2 + \alpha^t \langle \delta_{\mathbf{w}}^t, K_{\mathbf{w}}^t \rangle + \alpha^t ||\delta_{\mathbf{w}}^t||_2^2
\end{aligned}$$

where the last inequality follows from Lemma 2 with $\mathbf{w} = \mathbf{w}^t, \mathbf{g}_1 = G_{\mathbf{w}}^t, \mathbf{g}_2 = \mathbf{e}^t \otimes \nabla_{\mathbf{w}} \mathscr{L}_\lambda(\mathbf{w}^t; \theta^t)$ and $\alpha = \alpha^t$. Take expectations on both sides and then we get:

$$\begin{aligned}
&\mathbb{E}[\mathscr{L}_\lambda(\mathbf{w}^{t+1}; \theta^t) - \mathscr{L}_\lambda(\mathbf{w}^t; \theta^t)] \;\le\; \mathbb{E}[(-\alpha^t + \frac{L(\alpha^t)^2}{2}) ||k_{\mathbf{w}}^t||_2^2] + \mathbb{E}[\alpha^t \langle \delta_{\mathbf{w}}^t, K_{\mathbf{w}}^t \rangle] + \mathbb{E}[\alpha^t ||\delta_{\mathbf{w}}^t||_2^2] \\
&\le \mathbb{E}[(-\alpha^t + \frac{L(\alpha^t)^2}{2}) ||k_{\mathbf{w}}^t||_2^2] + \alpha^t (\sigma^t)^2
\end{aligned}$$

where the last equality is due to $\mathbb{E}[\delta_{\mathbf{w}}^t] = 0$ and Eq. (2).

Similarly, when considering $\mathscr{L}_\lambda(\theta^{t+1}; \mathbf{w}^{t+1}) - \mathscr{L}_\lambda(\theta^t; \mathbf{w}^{t+1})$, we have:

$$\begin{aligned}
&\mathscr{L}_\lambda(\theta^{t+1}; \mathbf{w}^{t+1}) - \mathscr{L}_\lambda(\theta^t; \mathbf{w}^{t+1}) \;\le\; \langle \nabla_\theta \mathscr{L}_\lambda(\theta^t; \mathbf{w}^{t+1}), \theta^{t+1} - \theta^t \rangle + \frac{L}{2} ||\theta^{t+1} - \theta^t||_2^2 \\
&= -\alpha^t \langle K_\theta^t, k_\theta^t \rangle + \frac{L(\alpha^t)^2}{2} ||k_\theta^t||_2^2 \;=\; -\alpha^t \langle k_\theta^t, k_\theta^t \rangle + \frac{L(\alpha^t)^2}{2} ||k_\theta^t||_2^2 + \alpha^t \langle k_\theta^t - K_\theta^t, k_\theta^t \rangle \\
&= -\alpha^t \langle k_\theta^t, k_\theta^t \rangle + \frac{L(\alpha^t)^2}{2} ||k_\theta^t||_2^2 + \alpha^t \langle \delta_\theta^t, k_\theta^t \rangle \;=\; (-\alpha^t + \frac{L(\alpha^t)^2}{2}) ||k_\theta^t||_2^2 + \alpha^t \langle \delta_\theta^t, \delta_\theta^t \rangle + \alpha^t \langle \delta_\theta^t, K_\theta^t \rangle
\end{aligned}$$

Take expectations on both sides and then we get:

$$\mathbb{E}[\mathscr{L}_\lambda(\theta^{t+1};\mathbf{w}^{t+1}) - \mathscr{L}_\lambda(\theta^t;\mathbf{w}^{t+1})] \le \mathbb{E}[(-\alpha^t + \frac{L(\alpha^t)^2}{2})||k^t_\theta||_2^2] + \mathbb{E}[\alpha^t||\delta^t_\theta||_2^2] + \mathbb{E}[\alpha^t\langle\delta^t_\theta, K^t_\theta\rangle]$$
$$\le \mathbb{E}\big[(-\alpha^t + \frac{L(\alpha^t)^2}{2})||k^t_\theta||_2^2\big] + \alpha^t(\sigma^t)^2$$

Above all, we have:

$$\mathbb{E}[\mathscr{L}_\lambda(\theta^{t+1},\mathbf{w}^{t+1}) - \mathscr{L}_\lambda(\theta^t,\mathbf{w}^t)] = \mathbb{E}[\mathscr{L}_\lambda(\theta^{t+1},\mathbf{w}^{t+1}) - \mathscr{L}_\lambda(\theta^t,\mathbf{w}^{t+1})] + \mathbb{E}[\mathscr{L}_\lambda(\theta^t,\mathbf{w}^{t+1}) - \mathscr{L}_\lambda(\theta^t,\mathbf{w}^t)]$$
$$\le \mathbb{E}\big[(-\alpha^t + \frac{L(\alpha^t)^2}{2})(||k^t_\theta||_2^2 + ||k^t_\mathbf{w}||_2^2)\big] + 2\alpha^t(\sigma^t)^2$$

Then, because $0 < \alpha^t < \frac{2}{L}$, we have:

$$\alpha^t\mathbb{E}[||k^t_\theta||_2^2] + \alpha^t\mathbb{E}[||k^t_\mathbf{w}||_2^2] \le \frac{2}{2 - L\alpha^t}\mathbb{E}\big[\mathscr{L}_\lambda(\theta^t,\mathbf{w}^t) - \mathscr{L}_\lambda(\theta^{t+1},\mathbf{w}^{t+1}) + 2\alpha^t(\sigma^t)^2\big]$$

Summing the above inequality over t and using Eq. (4), we have

$$\sum_{t=1}^{\infty}\alpha^t\mathbb{E}[||\frac{(\theta^{t+1},\mathbf{w}^{t+1}) - (\theta^t,\mathbf{w}^t)}{\alpha^t}||_2^2] = \sum_{t=1}^{\infty}\alpha^t\mathbb{E}[||\frac{\theta^{t+1}-\theta^t}{\alpha^t}||_2^2] + \sum_{t=1}^{\infty}\alpha^t\mathbb{E}[||\frac{\mathbf{w}^{t+1}-\mathbf{w}^t}{\alpha^t}||_2^2]$$
$$= \sum_{t=1}^{\infty}\alpha^t\mathbb{E}[||k^t_\theta||_2^2] + \sum_{t=1}^{\infty}\alpha^t\mathbb{E}[||k^t_\mathbf{w}||_2^2] \le \infty$$

Hence, by Lemma 3, there must exist an index sub-sequence $\mathscr{M}$ such that

$$\lim_{\substack{t\to\infty\\ t\in\mathscr{M}}}\mathbb{E}||\frac{(\theta^{t+1},\mathbf{w}^{t+1}) - (\theta^t,\mathbf{w}^t)}{\alpha^t}||_2^2 = 0. \quad (10)$$

□

References

1. Ghadimi, S., Lan, G. & Zhang, H. Mini-batch stochastic approximation methods for nonconvex stochastic composite optimization. *Math. Program.* **155**, 267–305 (2016).
2. Xu, Y. & Yin, W. Block stochastic gradient iteration for convex and nonconvex optimization. *SIAM J. on Optim.* **25**, 1686–1716 (2015).
